# An SMS Text Message–Based Type 2 Diabetes Prevention Program for Hispanic Adolescents With Obesity: Qualitative Co-Design Process

**DOI:** 10.2196/46606

**Published:** 2023-08-02

**Authors:** Erica Soltero, Callie Lopez, Sandra Mihail, Ayleen Hernandez, Salma M Musaad, Teresia M O'Connor, Debbe Thompson

**Affiliations:** 1 USDA/ARS Children's Nutrition Research Center Department of Pediatrics Baylor College of Medicine Houston, TX United States

**Keywords:** Hispanic health, adolescents, digital health, physical activity, sleep, type 2 diabetes, mobile phone, smartphone

## Abstract

**Background:**

SMS text message–based interventions are a promising approach for reaching and engaging high-risk youths, such as Hispanic adolescents with obesity, in health promotion and disease prevention opportunities. This is particularly relevant, given that SMS text messaging is widely accessible and available and that adolescents are frequent texters. Including youths in the development of SMS text message content can lead to more acceptable and relevant messaging; however, few studies include this group as cocollaborators.

**Objective:**

This study aimed to use a co-design process to inform the development of SMS text messages that promote healthy physical activity (PA) and sleep behaviors among Hispanic adolescents with obesity.

**Methods:**

The co-design framework uses multiple methods across several phases. Self-determination theory and a literature review of SMS text message–based interventions guided the background and research phases. In the co-design phase, Hispanic adolescents (n=20) completed in-depth interviews to identify barriers and facilitators of PA and sleep, preferences for ways to emphasize key self-determination theory constructs (autonomy, competence, and relatedness), and suggestions for making SMS text message content engaging. In the design and content phase, interview findings were used to develop initial SMS text messages, which were then evaluated in the early evaluation phase by experts (n=6) and adolescents (n=6). Feedback from these panels was integrated into the SMS text message content during refinement.

**Results:**

The background phase revealed that few SMS text message–based interventions have included Hispanic adolescents. Common barriers and facilitators of activity and sleep as well as preferences for ways in which SMS text messages could provide autonomy, competence, and relatedness support were identified in the co-design phase. The youths also wanted feedback about goal attainment. Suggestions to make SMS text messages more engaging included using emojis, GIFs, and media. This information informed an initial bank of SMS text messages (N=116). Expert review indicated that all (116/116, 100%) SMS text messages were age and culturally appropriate; however, some (21/116, 18.1%) did not adequately address youth-identified barriers and facilitators of PA and sleep, whereas others (30/116, 25.9%) were not theoretically adherent. Adolescents reported that SMS text messages were easy to understand (116/116, 100%), provided the support needed for behavior change (103/116, 88.8%), and used mostly acceptable language (84/116, 72.4%). Feedback was used to refine and develop the final bank of 125 unique text messages.

**Conclusions:**

Using a co-design process, a theoretically grounded, appealing, and relevant bank of SMS text messages promoting healthy PA and sleep behaviors to adolescents was developed. The SMS text messages will be further evaluated in a pilot study to assess feasibility, acceptability, and preliminary efficacy. The co-design process used in this study provides a framework for future studies aimed at developing SMS text message–based strategies among high-risk adolescents.

**International Registered Report Identifier (IRRID):**

RR2-10.1016/j.cct.2023.107117

## Introduction

### Background

Lifestyle interventions focused on health behaviors such as physical activity (PA) and sleep remain as a first line of defense for the prevention of obesity-related diseases such as type 2 diabetes (T2D) [1]. SMS text messaging has been recognized as an important health promotion tool for engaging adolescents in lifestyle interventions [2,3]. Given the widespread availability and accessibility of SMS text message communication, strategies that leverage this method of communication are promising for reaching and engaging adolescents at high risk. For example, Hispanic youths are disproportionately burdened by high rates of obesity (27% vs 21.5% of the general population) and subsequent T2D compared with other pediatric subgroups [4,5]. SMS text message–based interventions may be ideal for this population, as 95% of Hispanic youths report owning their own mobile phone [6], and adolescents are the highest users of SMS text message communication compared with other age groups [3,7-9]. SMS text message–based strategies are cost-effective for participants because they are free to send or receive with most mobile phone plans used today, and an internet connection is not required [10]. In addition, they can be sent directly to participants across geographic boundaries. This can increase the reach of SMS text message–based interventions among adolescents and families by reducing the burden of common barriers. For example, adolescents and parents often report that barriers to participation in traditional in-person programs include lack of transportation, parent work schedules, and lack of childcare. Given that youth participation in in-person programs is often reliant on parents for transportation or availability, these factors limit participation in health promotion and disease prevention opportunities. Despite the potential of SMS text message–based interventions for overcoming these barriers, few such programs have been developed and tested among Hispanic adolescents [11-17].

Co-designing digital health interventions with the population of focus is critical for developing content that is feasible, engaging, and relevant [18,19]. The co-design process is rooted in participatory techniques that engage the end user from design to implementation [18-20]. Through a systematic process, participatory strategies may include qualitative and quantitative methods to understand and prioritize the needs and preferences of the end user. This information is then used to shape the content [21,22]. Given that SMS text messaging is the primary method of communication among adolescents, their input as co-designers of the content and implementation of SMS text message–based strategies has the potential to lead to more effective and sustainable behavior change [18]. However, few studies have engaged adolescents in the development process [18,23,24].

### Objective

The purpose of this paper is to describe the co-design process that informed the development of a bank of evidence-based SMS text messages to enhance PA and sleep for Hispanic adolescents with obesity. These messages, along with a Fitbit device, will be tested in a future T2D prevention intervention for this high-risk target group.

## Methods

### Overview

**Figure 1:**
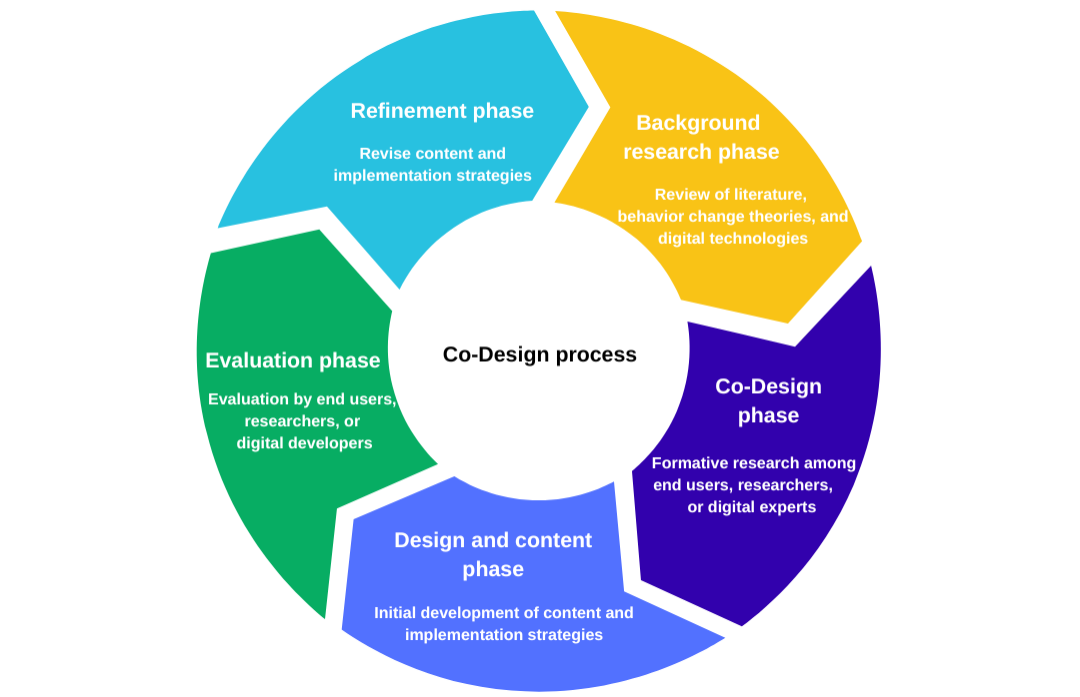
Co-design framework adapted from Beven et al [19], which is published under Creative Commons Attribution 4.0 International License [25].

The co-design framework used in this study was adapted from Bevan et al [19] (Figure 1). This framework allows for the use of multiple research methods applied across several phases among the population of interest and other stakeholders (eg, parents or teachers) [19,20]. This framework guides the assessment of acceptability, usability, and participant satisfaction while considering the context and anticipated use of the content created [19]. The 5 phases of the co-design process are described in the following sections.

### Phase 1: Background Research

The purpose of this first phase was 2-fold: (1) select a theoretical framework to ground the co-design process and (2) conduct a literature review of previous digital health interventions that have used SMS text messaging to promote health behaviors among adolescents. The investigative team selected the self-determination theory (SDT) as the guiding theoretical framework for this study, given that it is an age-appropriate and developmentally appropriate behavior change theory [26,27].

According to SDT, health behaviors become internalized and integrated into one’s sense of self (ie, autonomously motivated) as the basic psychological needs for autonomy (choice and control), competence (behavioral capability and mastery), and relatedness (connection to important others or one’s own self) are satisfied [28,29]. SDT contends that, as these psychological needs are satisfied in relation to a targeted behavioral outcome (eg, PA and sleep), performing that behavior becomes internalized and integrated into one’s sense of self, that is, it becomes autonomously motivated (ie, self-endorsed) [26-28,30].

In subsequent phases of the co-design process, this guiding theory will be used to ground SMS text messages in the SDT framework, in an effort to encourage self-endorsed adoption of these behaviors. The use of a guiding theoretical framework to develop SMS text message content has the potential to provide insight into the underlying theoretical mechanisms by which SMS text message content may drive sustainable changes in PA and sleep [31] and the pathway through which behavior change occurs.

The goal of the literature review was to identify the current state of the science on digital health interventions that use SMS text messaging to promote health behaviors among adolescents. The aim was to identify the strengths and weaknesses of the existing evidence about the use of SMS text messaging as a health promotion tool. The literature review highlighted gaps in the existing evidence base and opportunities to advance the science.

### Phase 2: Co-Design

In this phase, semistructured, in-depth interviews were conducted to identify the needs, preferences, and desired support for PA and sleep behaviors. A community sample of Hispanic adolescents (n=20) was recruited from partnering pediatric clinics, community organizations, and the Children’s Nutrition Research Center volunteer research database. Interested youths were screened via phone using the following inclusion criteria: self-identify as Hispanic or Latino origin, have obesity (BMI% ≥95th percentile and <120% of the 95th%), be aged between 14 and 16 years, and have own mobile phone. This age range was selected because adolescents in this age group have more autonomy and are more cognitively mature, making them more capable of acting upon SMS text message content and comprehending theoretically grounded content. Participants were excluded if they were taking a medication (ie, steroids) or diagnosed with a condition (ie, sleep apnea) that influences activity, sleep, or cognition.

A scripted guide consisting of open-ended questions was developed to elicit information about perceived facilitators of and barriers to PA and sleep, perceptions about psychological needs in relation to PA and sleep, self-endorsed personal values, preferences for goal setting, desired type of feedback about goal attainment, and preferences for texting style (eg, use of slang, emojis, and GIFs). Overall, 2 trained, bilingual research coordinators completed all interviews using the scripted interview guide. Interviews were scheduled at the convenience of the participant, conducted remotely using a private Zoom (Zoom Video Communications) link, and audio recorded. Participants were compensated. 

### Phase 3: Design and Content

In this phase, information gathered from qualitative interviews conducted in phase 2 was used to develop SMS text message content and an initial bank of SMS text messages. All SMS text messages were semipersonalized and designed to be ≤160 characters to increase readability and engagement, and a Fleschman-Kincade calculator was used to assess the readability [18]. All SMS text messages were modified and reworded until all the messages were written at or below the fourth-grade reading level. The research team iteratively reviewed and revised the initial bank of messages until key insights and feedback from in-depth interviews were incorporated. The initial bank created in this phase consisted of 116 SMS text messages.

### Phase 4: Early Evaluation

In this phase, 29.3% (34/116) of representative SMS text messages underwent expert review by panels consisting of researchers, adolescents, and parents. A subsample of 29.3% (34/116) of SMS text messages that represented key concepts from the co-design phase (eg, goal setting, feedback about goal attainment, and SDT construct support) was selected for review, given that SMS text message content followed a similar pattern from week to week, and there was concern that reviewing all the SMS text messages (116/116, 100%) would be too great a participant burden for the expert and youth panels.

The expert panel (n=6) consisted of researchers with expertise in health promotion among Hispanic adolescents and families, digital health interventions, qualitative research, and SDT. Expert panel members were asked to review the subsample of SMS text messages and provide feedback via a Qualtrics (Qualtrics International Incorporated) survey. The survey asked experts to ensure that all SMS text messages were age appropriate, culturally appropriate, and adherent to SDT and used evidence-based behavior change techniques to promote activity and sleep. Using random assignment, at least 2 experts were asked to review each SMS text message. After the review was completed, the panel convened in a 2-hour workshop to discuss feedback and provide additional suggestions for refinement.

A subsample of adolescents (n=6) from the background research phase was invited to serve on the youth panel and review and provide feedback about the same subsample of SMS text messages (34/116, 29.3%) reviewed by the expert panel. This group was asked to complete a Qualtrics feedback survey, where they were instructed to read each SMS text message and respond to a series of questions that assessed the usefulness of the tips and strategies shared, degree to which the desired behavior change techniques were integrated, perceptions about the goal-setting assistance provided, perceptions about the feedback on goal attainment provided, SMS text message format, and how accurately the participant perceives the SDT constructs. Feedback about SMS text message format included an assessment of readability, length, and style of language (ie, cool vs cringe). Using random assignment, at least 2 youth panel members were asked to review each SMS text message. After all feedback surveys were completed, a brief, scripted phone interview was conducted to clarify and gain a deep understanding of any survey responses that were unclear. The phone interview was also used to ask follow-up questions related to intervention implementation (eg, desired SMS text message frequency). Parents were asked to complete a separate survey focused on parental perceptions about an SMS text message–based health program in general, perceived barriers to program participation (ie, mobile phone ownership and data plans), and family norms and rules regarding SMS text messaging and phone use.

### Phase 5: Refinement

Using feedback and insights from adolescents, parents, and research experts in the early evaluation phase, the research team refined the existing SMS text message library and implementation strategies.

### Statistical Analyses

Audio recorded in-depth interviews from phase 2 (co-design phase) were transcribed verbatim and reviewed for accuracy before coding. A priori codes were identified before coding (ie, deductive coding approach). Codes were defined and recorded in a codebook. Using the codebook, 2 trained coders independently coded the transcripts and met routinely to compare codes and discuss and resolve any differences. NVivo (version 9; QSR International) was used to facilitate coding. Content analysis was performed to identify barriers to and facilitators of activity and sleep behaviors, desired behavior change techniques, and SDT-specific needs support [32]. Descriptive analyses were conducted using SPSS to examine the frequency with which codes were used and to analyze survey results obtained from experts, adolescents, and parents.

### Ethics Approval

Ethics approval was obtained from the institutional review board at Baylor College of Medicine (H-50331).

### Consent for Participation

Participants and their parents received an electronic copy of the informed consent form via email or SMs text message, and the consent was further reviewed by a trained research team member over the phone or via Zoom. Parents provided written electronic informed consent, and adolescents provided written assent using REDCap (Research Electronic Data Capture; Vanderbilt University) [33].

### Data Management

Once consent and assent were obtained, participants were assigned a unique numerical identifier. A restricted-access, password-protected study key of names and identifiers was saved to our institute’s secure drive. Interview transcripts were deidentified, and participant names were replaced with each participant’s unique identifier to provide privacy and confidentiality. All data were collected using our institution’s Zoom and REDCap accounts and stored on the institution’s secure drive.

### Compensation

Participants received US $25 as compensation for their time and expertise in completing the in-depth interview and an additional US $25 for reviewing and providing feedback about SMS text messages.

## Results

### Overview

Participants’ demographic characteristics are shown in Table 1. In total, 20 Hispanic adolescent girls (n=11, 55%) and boys (n=9, 45%) participated in in-depth interviews, which lasted between 40 and 70 minutes. Participants were predominantly from lower socioeconomic backgrounds and most (17/20, 85%) identified as Mexican American. Male participants were more likely to come from female-headed or separated households, and the responding parents of male participants were more likely to be employed.

**Table 1:** Demographic characteristics.

Variables		Adolescent girls (n=11)	Adolescent boys (n=9)
Age (years), mean (SD)		15.1 (0.9)	15.2 (0.7)
BMI (kg/m^2^), mean (SD)		37.3 (7.8)	35.7 (4.9)
**Hispanic group, n (%)**			
	Mexican	10 (91)	7 (78)
	Salvadoran	0 (0)	2 (22)
	Honduran	1 (9)	0 (0)
**Living status, n (%)**			
	2-parent household	7 (64)	3 (33)
	Female-headed household	2 (18)	3 (33)
	Separated parents	2 (18)	2 (22)
	Other	0 (0)	1 (11)
**Level of education, n (%)**			
	Sixth grade or less	1 (9)	1 (11)
	Eighth grade or less	2 (18)	1 (11)
	Some high school	3 (27)	3 (33)
	High school or equivalent	3 (27)	1 (11)
	Some college	2 (18)	1 (11)
	Technical school	0 (0)	1 (11)
	Postgraduate study	0 (0)	1 (11)
**Employment, n (%)**			
	Yes	4 (36)	8 (89)
	No	7 (64)	1 (11)
**Household income (US $), n (%)**			
	<10,000	1 (9)	0 (0)
	10,000-19,999	2 (18)	1 (11)
	20,000-29,999	3 (27)	2 (22)
	30,000-39,999	3 (27)	2 (22)
	40,000-49,999	1 (9)	2 (22)
	50,000-59,999	1 (9)	1 (11)
	60,000-69,999	0 (0)	0 (0)
	70,000-79,999	0 (0)	1 (11)

### Phase 1: Background Research

Background research revealed several population, methodology, and design gaps in the literature on the use of SMS text messaging to promote health behaviors among adolescents. To start, few studies have focused on this group, particularly those at high risk such as Hispanic adolescents [2,34]. Most studies so far have focused on young age groups or adult populations [2,18]. In addition, there has been a lack of studies that have used SMS text messaging in conjunction with a personal activity tracker such as a Fitbit device among adolescents [2,12,35]. There is an overall lack of feasibility studies, and few studies have examined the independent effects of SMS text messaging on outcomes of interest [2]. Common behavior change techniques reported in the available studies include providing information, skills, solutions for barriers, feedback, goal setting, and self-monitoring [2,18,34]. Finally, few studies found in the literature so far have reported the use of a behavior change theory to guide the integration of behavior change techniques and theoretical constructs into SMS text message–based interventions [2,18]. We also found that few studies have used a multibehavioral approach, as many have focused solely on 1 behavior such as PA or sleep [2,18,34,36].

Our review of the literature provided a strong justification for our focus population, inclusion of sleep as a behavioral target, and use of a strong theoretical framework. The SMS text messages will be used in conjunction with a Fitbit Charge 5 device, given that this approach has proven to be the most effective among adults [37,38]. However, on the basis of background findings, we will give adolescents in the waitlist control arm a Fitbit device without SMS text messages to examine the additional contributions of SMS text messages beyond device use only. The behavior change techniques noted in our review were applied to SMS text message content based on guidance from the adolescents and findings from the co-design phase.

### Phase 2: Co-Design

#### Overview

This section provides an overview of patterns and insights gained from the qualitative interviews conducted in the co-design phase. We have presented exemplar quotes that support the identified patterns and insights regarding barriers to and facilitators of PA and sleep, desired competence, autonomy, relatedness support, and desired feedback about goal attainment and SMS text message elements for engaging this age group. To provide context, quotes are identified by sex, and each quote presented is from a different participant.

#### PA Barriers

When asked about barriers to PA, participants mentioned screen time (12/20, 60%), school (9/20, 45%), household chores (5/20, 25%), and lack of support (5/20, 25%):

...It’s like starting to get dark now, like really early. I don’t have time to do exercise, because of both my homework and early it’s becoming dark.

Adolescent female participant

Having seen my phone...I just choose to, like, focus on that and avoid, you know, being active.

Adolescent female participant

#### Facilitators of PA

Approximately half of the adolescents identified family (12/20, 60%) and friend support (9/20, 45%) and concern regarding their future health (4/20, 20%) as factors that encourage them to be physically active:

My friend, one of my close friends like he got like really skinny over the summer. And he like, motivates me sometimes to do stuff...like, go like on jogs, and like start working out and stuff.

Adolescent male participant

My family, cause when I know that they believe in me...they cheer me on. And I have more confidence for that.

Adolescent female participant

#### Sleep Barriers

Participants expressed that common barriers to sleep included excessive screen time (17/20, 85%), general noises in the home or community environment (10/20, 50%; ie, barking dogs, street traffic, and family members), and staying up late to play video games (6/20, 30%):

I’m the one who does sleep against the wall where my dogs are...that doesn’t let me sleep because they are barking most of the time at night.

Adolescent female participant

My phone...I just want to keep watching videos...that’s why I stay up.

Adolescent male participant

#### Facilitators of Sleep

When questioned about facilitators of sleep, the adolescents discussed that having an active day (6/20, 30%) leads to better-quality sleep. They also discussed having a sleep routine (5/20, 25%) that includes activities such as taking a warm shower as important for facilitating sleep. In addition, the home sleep environment (4/20, 20%), which was described as having no lights, no background noise (eg, white noise), or a comfortable room temperature, was mentioned. Similarly, a quiet neighborhood (4/20, 20%), described as “safe” or “peaceful,” was also identified as a facilitator:

Probably that it’s quiet like outside...the community’s been very quiet here, so that that’s one big thing that helps.

Adolescent female participant

...After I finish showering my body just feels able to like rest itself...It’s just for me, I have to be like, clean to go to bed...I feel a little more relaxed.

Adolescent male participant

#### Competence Support Needed for Improving PA

When asked about the information, knowledge, or skills that would encourage PA, participants responded that they desired SMS text message reminders to be active (17/20, 85%), messages that were encouraging and used motivating words (14/20, 70%), suggestions for different types of activities that they could do (8/20, 40%), and feedback about their progress toward goals (5/20, 25%):

I would say any reminder that was set sometimes, or like a timer, that could like, give me memory, or to remind me, that I need to do something and be more active...

Adolescent female participant

I would like to receive something that keeps me interested so that I can do it every week. Like saying “Oh, nice job man!” and “now are you ready for this next week?”...And then if you did a bad job ummm...you can say something like “keep your head up, champ.”

Adolescent male participant

#### Competence Support Needed for Improving Sleep

When asked about the information, knowledge, and skills that would encourage them to get more sleep, participants responded that they desired SMs text messages that included reminders to go to bed and reminders to stop using screen devices such as their phone or video games (5/20, 25%). They also expressed a desire for facts about sleep and health (4/20, 20%) and recommendations for achieving better sleep (3/20, 15%):

Tik tok has these little, like, little videos, or whatever that says, like, “Hey, you’ve been scrolling for too long.”...I guess adding something like it...like if you see this text, you’re either wasting your time on your phone or not asleep.

Adolescent female participant

Just give them like a fact or something...you definitely could put something in there that’s like, “hey, you should watch out for this. Because if you don’t, you’re going to get this. And I know you don’t want to get this.”

Adolescent male participant

#### Autonomy Support Needed for Changing Health Behaviors

To inform SMS text message content focused on building autonomy, adolescents were asked how they desired to be encouraged to take charge of their health. Participants responded that we could encourage adolescents to make decisions and exercise control over their health behaviors by emphasizing how important it is to have a healthy lifestyle (9/20, 45%), providing guidance about how they can take charge of their own health (5/20, 25%), and providing motivation and encouragement for adolescents to take charge of their health (4/20, 20%):

It [making health decisions] can be a little too much sometimes for maybe well my age to like take care of their health. It’s just better to have an adult even if it’s just to like go, “Do you think this is okay?”

Adolescent female participant

Tell them the risk of not taking care of their- taking charge of their health. The pros and cons.

Adolescent male participant

#### Self-Endorsed Personal Values

To inform relatedness SMS text messages that connect goal achievement to a personal value, a list of 12 values was presented, and each participant was asked to identify the top 3 values that were most important to them [39]. The top values consistently identified among the adolescents were being a good person (10/20, 50%), being healthy and fit (11/20, 55%), being successful (9/20, 45%), and getting good grades (8/20, 40%):

That value [being healthy and fit] is important to me because...I had a grandfather, who was a diabetic...And then the doctors would suggest that, like, “hey, we suggest that you exercise, try to stay fit,” and he would do it for like, two days, and then he’ll go back to eating bad.

Adolescent female participant

Because I like to succeed at what I’m doing, like when I’m working out, lifting pretty good and like seeing my grades be good too, but in general, just being successful and seeing that I’m doing pretty good in life...

Adolescent male participant

#### Desired Feedback About Goal Attainment

Participants were asked to describe the type of feedback that they would like to receive about goal attainment. The adolescents felt that SMS text messages providing feedback should include positive words when goals are met (11/20, 55%). Regardless of goal attainment, they recommended that we avoid all criticism (12/20, 60%). When goals are not met, they suggested that we use gentle words to let youths know that they did not meet their goal (5/20, 25%) and encouraging words that motivate youths to try hard next week (6/20, 30%):

I guess how you would get like a message from like, your iPhone for like your screen time where it’s like, “Hey, you cut down on this today, or this week, and like, here’s your percentages,” and it provides you like with the graph, and then shows your progress...and then you have like a what is it “Keep up the good work!” or something like that or some encouraging message.

Adolescent male participant

When discussing the type of feedback that was not desired, a participant shared the following:

Some sort of like, “Oh, you didn’t accomplish it” or “Good try, good job this week, but try better” message like that, or messages that sort of talk, sort of criticizes their work or them trying.

Adolescent male participant

#### Engaging Adolescents via SMS Text Messages

When asked about how to design SMS text messages that were engaging for adolescents, the participants suggested the use of emojis (13/20, 65%), GIFs (12/20, 60%), pictures (10/20, 50%), and video links (9/20, 45%):

I think that just by using those [emojis, GIFs, and pictures], it would make the text less formal. It gives you a sense of comfort and trusting. So it motivates you...

Adolescent female participant

### Phase 3: Design and Content

**Figure 2:**
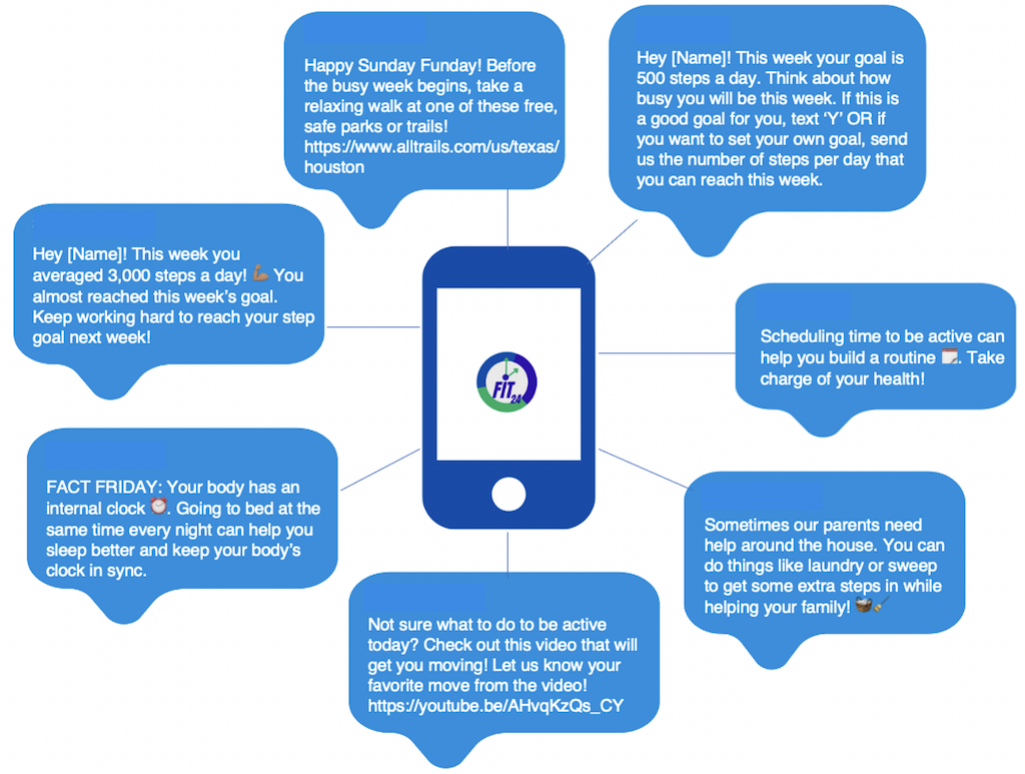
Sample weekly SMS text message schedule.

Figure 2 represents examples of how qualitative patterns and insights from the background research phase on how adolescents would like to receive and use SMS text messages were translated into initial SMS text messages content. First, a weekly schedule for implementing and sending SMS text messages was developed. SMS text messages to be sent on Monday were designed to assist adolescents in setting new PA and sleep goals for the week. Goals will be adaptive—that is, on the basis of the previous week’s step and sleep attainment, so that goals for each behavior increase successively across weeks [40]. To do this, Fitbit data will be integrated into a 2-way, Health Insurance Portability and Accountability Act–secure, SMS text message–based platform developed for research studies (Mosio). The following formulas were developed in our Mosio SMS text message platform to provide goal-setting assistance:

1. Step goal
  = (average steps/day from Monday to Friday) + (10% of average steps/day from Monday to Friday)
2. Sleep goal
  = (average sleep hours/night from Monday to Friday) + (10% of average sleep hours/night from Monday to Friday)

SMS text messages sent throughout the week (Tuesday-Thursday) will provide general tips and strategies to provide the type of competence and autonomy support desired by adolescents. In addition to goal setting, desired evidence-based, behavior change techniques such as self-monitoring, behavioral prompts or reminders, and tips and strategies for overcoming identified barriers to and facilitators of activity and sleep will be sent to adolescents from Tuesday to Thursday. SMS text messages were segmented into straightforward prompts or reminders to be active or go to sleep and prompts for activity that provide actionable suggestions and strategies, given that the adolescents expressed a need for guidance about how to be more active, how to get better sleep, and how to take charge of their health. To satisfy the desire for health facts and information, the group received a “Fact Friday” SMS text message every Friday that provided health information and facts. SMS text messages were configured such that every Saturday, they will receive feedback about goal attainment. Relatedness SMS text messages accompanied feedback texts to connect goal attainment to a self-endorsed personal value. SMS text messages scheduled for Sunday will include a “Sunday Funday” suggestion for doing a fun and engaging activity with a family member or friend.

SMS text message content was also developed to support basic psychological needs. For example, goal-setting SMS text messages were written in a manner that considers the participant’s autonomy, by giving the participant choice and control over setting their activity and sleep goals and choice and control in how they achieve their activity and sleep goals. Autonomy support was also provided by wording SMS text messages in a manner in which recommended activities and behavior change strategies are offered as suggestions, as opposed to commands that the adolescents are obligated to fulfill. Competence support was provided as SMS text messages that were worded to provide structure for mastery experiences for engaging in activity or sleep. These SMS text messages included desired behavior change techniques such as prompts or reminders to self-monitor or check progress toward activity and sleep goals. Relatedness-based SMS text messages were written in a manner that focused on connecting goal attainment to a self-endorsed value, so that the behavior becomes internalized.

In response to design elements desired by the adolescents to increase engagement, 3 infographic images were designed and 4 short videos were created to provide suggestions for activities and information such as establishing a sleep routine. Links to the videos were integrated into SMS text messages and easily accessible via a private YouTube study page, and images were embedded into SMS text message content when applicable.

### Phase 4: Early Evaluation

Among research experts, there was 100% agreement by the experts that all SMS text messages were age appropriate and culturally appropriate. Of the 34 SMS text messages reviewed, the experts felt that 6 (18%) did not adequately address barriers to and facilitators to PA and sleep, 6 (18%) did not adequately reflect behavior change techniques desired by the adolescents, and 9 (26%) were not theoretically adherent (ie, did not provide competence support as intended). Experts provided recommendations for making the SMS text messages more concise, suggested greater use of emojis, and recommended the removal of any PA tips or suggestions that required potentially costly items (eg, bikes).

After reviewing the subsample of SMS text messages, the adolescents felt that they accurately represented values of high importance to them (32/34, 94%), that they would try the suggested activities (30/34, 88%), and that the SMs text messages would be helpful for meeting their goals (30/34, 88%). They mostly felt that the language used was cool (24/34, 71%) as opposed to “cringe” and that the length of the messages was mostly “just right” (28/34, 82%) as opposed to very short or very long. Most participants (5/6, 83%) reported that the wording in the SMS text messages was encouraging and that all the SMS text messages were clear and easy to understand (34/34, 100%). In follow-up phone interviews, 66% (4/6) of adolescents expressed that they desired assistance in setting behavior goals. A frequency of 2 to 3 SMS text messages per day was acceptable to most adolescents (5/6, 83%). Although 50% (3/6) of the group felt comfortable in sharing SMS text messages with a friend or family member, 50% (3/6) expressed that they would only like to share goals for accountability or receive instrumental support by having a friend or family member to be active with them.

Survey findings from parents (n=10) revealed that most parents (n=9, 90%) are interested in having their teenager participate in this type of program. Most parents (9/10, 90%) said that their teenager owns their own smartphone or mobile device, and approximately half had rules regarding phone use at home (6/10, 60%) and were knowledgeable about school rules regarding phone use at school (6/10, 60%). When asked about their desired involvement in the program, many parents (8/10, 80%) reported that they would also like to receive the SMS text messages being sent to their teenager. However, although parents desired to receive SMS text messages to stay informed about the program, some (4/10, 40%) felt that their teenager should participate in the program independently.

### Phase 5: Refinement

Using expert, youth, and parent feedback as a guide, SMS text messages were refined to be clearer and more concise. This included simplifying and providing more clarity about SMS text messages that experts felt were not theoretically adherent, did not adequately address barriers and facilitators, or did not appropriately use the intended behavior change technique. For example, SMS text messages viewed as “cringe” (ie, not appealing) were revised according to youth feedback and suggestions, and those identified as “too long” were reworded to be short. We also removed any wording that could be considered as medical or research jargon [19]. SMS text messages were also reviewed to remove any mention to pay-for-use or costly resources that may not be broadly accessible to our population (eg, bikes and gym use).

Expert, youth, and parent feedback was also used to develop an implementation strategy. To decrease the risk of adolescents feeling overwhelmed by having both activity and sleep goals, the investigative team decided to focus solely on the PA goal for the first 4 weeks of the study before adding the sleep goal. On the basis of youth and parent feedback, SMS text messages were scheduled to be sent before (8 AM) and after (4 PM) school. Regarding overall SMS text message structure, we developed 12 focus areas, 1 for each week of the intervention [40]. Each theme addressed 1 to 2 categories from in-depth interviews such as screen time, setting a sleep environment, and making a plan for getting active. SMS text messages were structured to develop a storyline, which is a series of scheduled and automated SMS text messages within the Mosio platform. The final library consisted of 125 unique SMS text messages.

## Discussion

### Principal Findings

Digital health solutions such as SMS text message–based interventions offer promise as accessible, affordable, and potentially scalable strategies for health promotion and disease prevention efforts [18]. This study used a co-design approach to develop a library of theoretically grounded SMS text messages to promote healthy activity and sleep behaviors among Hispanic youths with obesity. The qualitative and quantitative data gathered from Hispanic adolescents, parents, and research experts led to the development of an SMS text message library that addresses barriers and leverages facilitators of PA and sleep in a manner that satisfies Hispanic adolescents’s basic psychological needs (autonomy, competence, and relatedness), while tailoring SMS text messages to the design preferences of this age group.

Adolescence is a life period in which young people begin to establish autonomy and desire more independence in making health decisions [41]. However, in this study, the adolescents expressed a need for recommendations about how to take charge of their health and desired assistance in setting behavioral goals. This is consistent with previous studies demonstrating that adolescents may not yet have the health knowledge or skills needed to autonomously set optimal behavioral goals [39,42,43]. To provide autonomy support, strategies and suggestions for improving activity and sleep were framed to give adolescents choice by using phrases such as “you can try” or “this is just one example you can choose.” This type of age-appropriate autonomy support has been shown to enhance their enjoyment as they are presented with different PAs [44,45]. To guide this group with goal setting while providing autonomy support, the research team developed SMS text messages that suggest a weekly step and sleep goal before giving adolescents the opportunity to choose the suggested goal or set their own goal. This approach is based on a key premise in SDT (ie, providing choice and control) and is a necessary component for enhancing the basic psychological need for autonomy. Previous studies demonstrated that having choice or control over goals can lead to a long-term commitment to behavior change [46] as the regulation of the behaviors becomes perceived as one’s own choice and becomes internalized [47,48].

The adolescents desired multiple behavior change techniques that are consistent with the current digital health literature and SDT perspectives about competence support. Through SMS text messages, adolescents will practice healthy decision-making, self-monitor progress toward goals, and receive tips and strategies for increasing PA and sleep. These techniques can provide them with mastery experiences as they develop the skills needed to achieve behavioral goals, thereby enhancing the basic psychological need for competence [49]. Prompts and reminders to engage in behaviors were desired by adolescents. On some occasions, the adolescents referenced the types of prompts and reminders that they experience on familiar social media apps such as TikTok’s prompt to stop scrolling after long periods. Given the level of exposure to technology at such a young age, today’s adolescents are very familiar with push notifications, which may act as reminders or prompts sent by one’s smartphone [50]. These types of notifications instantaneously deliver information to the participant and have been shown to increase user engagement in SMS text messaging [51]. Although the group desired reminders to engage in behaviors, such as prompts to get off their phone or to go to sleep, evidence about the use of these types of prompts for behavior change is inconclusive [52,53]. Some studies have reported that prompts are effective for behavior change, whereas other studies have found that adolescents may experience feelings of guilt if they are unresponsive to prompts or they may even experience “prompt fatigue,” all of which can reduce feelings of competence [54]. In addition to simply receiving a behavioral prompt or reminder, it is clear from our evidence that adolescents also desire reminders and prompts that are more directive in nature and provide actionable suggestions or helpful information that can drive behavior change. Overall, our findings indicate that prompts should be delivered as notifications and contain more informative messages, and more studies are needed to understand the use of behavior prompts among adolescents.

In providing feedback about goal attainment, SMS text messages were worded in a manner that emphasized support, meaning that SMS text messages focused on acknowledging the effort and progress toward goals as opposed to a focus on performance (ie, success or failure). Providing encouraging and supportive feedback can enhance autonomous motivation, as it affirms the youth’s competence toward achieving behavior goals [55]. In response to adolescents’s concerns about future health and their desire for health facts, SMS text messages were developed to provide health facts that educate them about T2D and emphasize the importance of a healthy lifestyle for reducing their risk for diabetes. Often, future health concerns were centered on the experiences of family members or known others with T2D, meaning that adolescents were concerned that they would progress toward a diseased state and experience some of the complications or hardships that they have observed in others (eg, pricking my finger every day). Providing a rationale for behavior change that is valued by participants, such as future health, is another way of fostering competence [56]. The high heritability of T2D among Hispanic families and the findings from our formative study warrant further exploration of the intergenerational impact of T2D within Hispanic families and how these concerns might be leveraged in future disease prevention efforts.

According to SDT, satisfying the need for relatedness is essential for adolescents to fully endorse activity and sleep behaviors [57], and this has been demonstrated in multiple studies [58,59]. One of the ways in which relatedness is operationalized in this study is through feedback SMS text messages that connect goal attainment to a personal value commonly endorsed among Hispanic adolescents in this study (eg, being active because one values the health benefits), a strategy that has been shown to lead to increased PA [39]. This approach allows adolescents to feel that their decisions are self-endorsed, rather than demanded by others, which can result in a greater likelihood of initiating and maintaining the behavior [57]. Relatedness through social connections with others will also be fostered through SMS text messages that encourage adolescents to share progress on behavior goals or engage in an activity with friends or family. Feeling connected to others and having a sense of belonging with others who engage in the activity with them can increase one’s desire to engage in that behavior. This may be a particularly important intervention input, given that familism or *familia* is an important cultural value among Hispanic youths and families [60].

Additional insights obtained through interviews and surveys yielded novel information to guide the implementation of the forthcoming SMS text message–based intervention. The adolescents suggested the use of emojis, GIFs, and media to make SMS text messages engaging. Other studies have also reported a desire among adolescents for emojis and media [36,61] and have found that the use of these in SMS text messages can increase participant engagement [62,63]. Engagement is an important consideration, given that previous SMS text message–based interventions in other high-risk, hard-to-reach populations has been very high [64]. There is little information in the literature about the timing and frequency of SMS text messages [10]; however, input from adolescents revealed that 2 to 3 SMS text messages per day were acceptable and should be sent before (8 AM) and after (4 PM) school to adhere to parent and school rules about phone use.

### Conclusions

This study describes the use of the co-design process to develop SMS text message content that is acceptable and engaging and meets the developmental and contextual needs of Hispanic adolescents with obesity to promote healthy PA and sleep behaviors. In addition to guiding SMS text message content, the formative, qualitative, and quantitative data collected through the co-design process provided key insights into factors that influence the implementation of SMS text–based strategies such as the timing and frequency of messages. This study may serve as a framework for future studies aiming to develop SMS text message–based interventions for pediatric populations at high risk. The SMS text message content developed in this study will undergo further evaluation as it will be tested in a forthcoming randomized, feasibility pilot study among Hispanic adolescents with obesity.

## Abbreviations

PA: physical activity
REDCap: Research Electronic Data Capture
SDT: self-determination theory
T2D: type 2 diabetes

## Data Availability

The data sets generated and analyzed during this study are not publicly available because of institutional ethics guidelines and the constituents of the approved ethics application. The data generated may be made available from the first author (ES) conditional upon approval from our institution’s ethics board.
